# MaxEnt-based suitable areas analysis of global lumpy skin disease under current and future climate scenarios

**DOI:** 10.3389/fmicb.2026.1816805

**Published:** 2026-09-07

**Authors:** Yanqun Wei, Zhenhuan He, Wenjing Li, Jingshan Bi, Zhongyi Zhao, Jialiang Xin, Rong Yang, Zhengji Wei, Mengjia Yang, Jing Huang, Jiangtao Zhang, Haotian Xue, Zhou Chen, Jiafang Xu, Jiafeng Ding, Min Zheng

**Affiliations:** 1 Guangxi Center for Animal Disease Control and Prevention, Nanning, China; 2 Beihai Center for Animal Disease Control and Prevention, Beihai, China; 3 Guangxi Agricultural and Animal Husbandry Engineering School, Liuzhou, China; 4 College of Animal Science and Technology, Guangxi University, Nanning, China; 5 Guangxi Agricultural Engineering Vocational Technical College, Fusui, China; 6 College of Veterinary Medicine, Sichuan Agricultural University, Chengdu, China

**Keywords:** environmental variable, future climate scenario, lumpy skin disease, maximum entropy model, *Stomoxys calcitrans*

## Abstract

**Introduction:**

Lumpy skin disease is an acute and subacute systemic infectious disease of cattle and buffalo.

**Methods:**

This study aimed to map the environmental suitable areas of lumpy skin disease under current and future climate scenarios using the MaxEnt model. Environmental variables were screened to construct distribution models for lumpy skin disease and its vector *Stomoxys calcitrans*. Then, their overlapping suitable areas were overlapped to identify highly suitable areas.

**Results:**

Key environmental variables influencing the environmental suitability of lumpy skin disease include cattle and buffalo distribution densities, the maximum temperature of the warmest month, and annual precipitation. Model performance was evaluated using AUC, TSS, and test omission rate, indicating good predictive ability. Under the future climate scenarios, climatically suitable areas for lumpy skin disease expanded, primarily in mid- to high-latitude regions between 40º and 70º, including the south-central U.S., northeastern Argentina, southeastern Brazil, Europe (excluding northern Europe, such as Iceland, Norway, and Sweden), the Mediterranean coastal areas of North Africa (such as Tunisia, northern Algeria, and Morocco), Turkey, Tajikistan, northern India, eastern China, South Korea, central and southern Japan, northern Indochina, and eastern Australia.

**Discussion:**

These results represent environmental suitability rather than actual disease risk, which also depends on factors such as animal movement, trade, vaccination, and surveillance. These findings offer valuable insights for the prevention and control of lumpy skin disease, mitigating both the suitable areas of disease occurrence and associated economic losses.

## 1 Introduction

Lumpy skin disease is a highly contagious vector-borne viral disease that primarily affects cattle and buffalo, caused by the lumpy skin disease virus (LSDV), a member of the Poxviridae family (Sprygin et al., 2022). It is classified as a notifiable disease by the World Organisation for Animal Health (WOAH), characterized by high morbidity, primarily affecting large ruminants (Manjunatha Reddy et al., 2023). Lumpy skin disease remains one of the most prominent viral diseases affecting cattle worldwide (Hamdi et al., 2021). Initially confined to sub-Saharan Africa, the disease has spread globally due to increased international trade in livestock and animal products, causing substantial economic losses, particularly in regions like the Middle East, Europe, and Asia (Wilhelm and Ward, 2023; Modethed et al., 2024). While several livestock diseases are influenced by climate and vector dynamics, lumpy skin disease stands out as a particularly important subject for climate suitability modeling due to its rapid expansion, strict vector dependence, and the relative scarcity of predictive studies.

The LSDV is typically transmitted through blood-sucking insects, contaminated feed, water, saliva, nasal secretions (Magori-Cohen et al., 2012; Liang et al., 2022). Therein, blood-sucking arthropods are the primary vectors for short-distance transmission of LSDV (El-Ansary et al., 2022). *Stomoxys calcitrans*, a vector of multiple domestic animal and human diseases, is identified as the principal transmitter of the virus (Gubbins, 2019; El-Ansary et al., 2022; Liang et al., 2022). Recent studies have highlighted a strong correlation between the prevalence of lumpy skin disease and periods of high rainfall, which boost insect activity (Ochwo et al., 2019). Taylor emphasized the influence of temperature and precipitation on the seasonal distribution of *S. calcitrans* (Taylor et al., 2007). Unlike directly transmitted livestock diseases (e.g., foot-and-mouth disease) whose spread is driven primarily by animal movement, lumpy skin disease transmission is tightly coupled to the activity and distribution of its arthropod vectors, which are directly modulated by climatic factors. This strong mechanistic link between climate and disease occurrence makes lumpy skin disease particularly well-suited for ecological niche modeling approaches. As such, understanding the relationship between lumpy skin disease occurrence, climate change, and the distribution of *S. calcitrans* is crucial for predicting potential suitable areas and supporting early warning and surveillance of the disease.

The Ecological Niche Model (ENM) can assess the potential environmental suitability of a research subject (such as a disease, pathogen, or species) by distribution location data with environmental variable data relevant to its occurrence or survival (Xu et al., 2025). The ENM has been widely utilized to predict specific environmental variables related to disease risk areas (Warren and Seifert, 2011; Dicko et al., 2014). The maximum entropy (MaxEnt) model is a commonly employed ENM for assessing species distribution and disease environmental suitability (He et al., 2024). This model leverages existing distribution data and environmental variables to evaluate the impact of various environmental variables on disease incidence and investigate the environmental suitability of disease (Ma et al., 2023). Although ENM approaches have been applied to other vector-borne livestock diseases such as bluetongue, their application to lumpy skin disease remains limited, especially at the global scale. To investigate the potential distribution of lumpy skin disease and its vector *S. calcitrans*, the MaxEnt model was employed, yielding reliable results.

In this study, the data of global lumpy skin disease outbreaks and *S. calcitrans* distribution were collected and processed, environmental variables were screened, and the MaxEnt model was built and evaluated to determine the environmentally suitable areas of lumpy skin disease. These results can provide valuable insights for targeted surveillance and early warning, supporting proactive planning for disease prevention.

## 2 Materials and methods

### 2.1 Processing of the data of global lumpy skin disease outbreaks and *S. calcitrans* distribution

**Figure 1:**
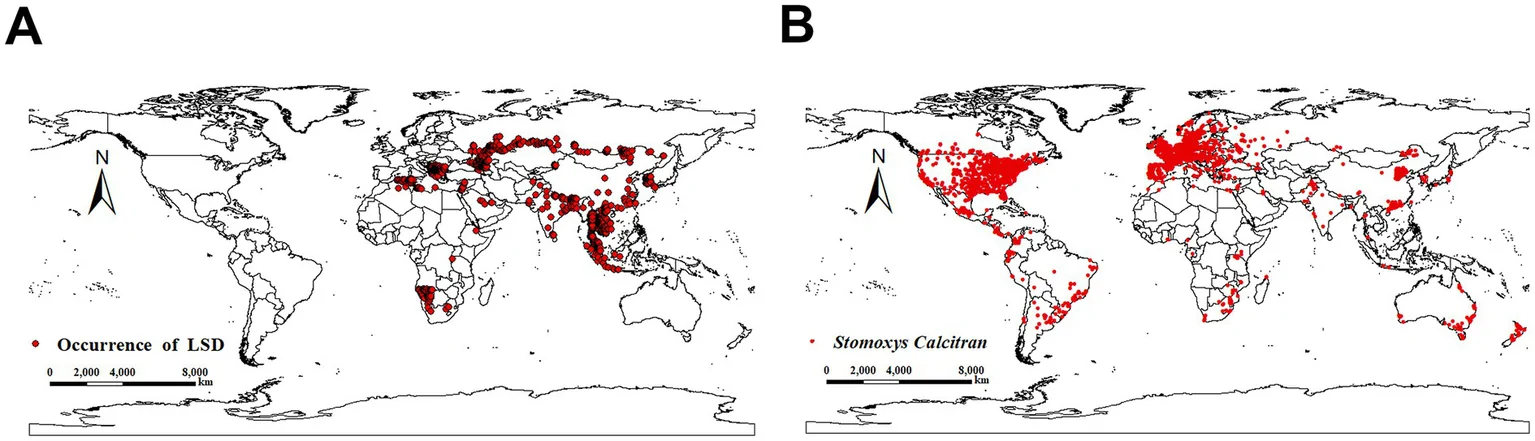
Global lumpy skin disease cases and *S. calcitrans* distribution from the year 2016 January to year 2025 July. **(A)** The 2,591 global occurrence sites of lumpy skin disease cases from the year 2016 January to year 2025 July. **(B)** The 4,000 global points of *S. calcitrans* distribution from the year 2016 January to year 2025 July.

The global data of 4,100 lumpy skin disease outbreaks from January 2016 to July 2025 was obtained through the United Nations Food and Agriculture Organization (FAO) International Animal Disease Information System (EMPRES-i, Downloaded in July 2025). The global data of 5,101 *S. calcitrans* distribution from January 2016 to July 2025 was obtained from the Global Biodiversity Information Facility (GBIF).^1^ The ENMTools software was utilized to reduce spatial autocorrelation and ensure the predictive performance of the model (Mazloum et al., 2023), ultimately retaining 2,591 records of the lumpy skin disease cases and 4,000 records of the *S. calcitrans* distribution, as shown in Figure 1.

### 2.2 Processing the environmental variables associated with lumpy skin disease and *S. calcitrans*

Data of 19 environmental variables of 2.5 arcminutes were obtained from the Worldclim 2.0 database.^2^ Besides, the data of cattle and buffalo density was obtained from the Food and Agriculture Organization.^3^

The correlation coefficient (r)-variable contribution rate (CVC) method was utilized to detect and screen multicollinearity among variables, and a high correlation between two variables was recognized as |r| ≥ 0.8 (Xu et al., 2017). Spearman correlation analysis was performed on 19 bioclimatic variables using SPSS software (IBM SPSS Statistics V22.0). The correlation coefficients between 19 environmental variables were calculated using the ENMTools software. Then, a pre-model was built to obtain the contribution rates of each environmental variable using these 19 environmental variables as a set of data. Retain the variables with |r| < 0.8, and if |r| ≥ 0.8, the variable with the lower contribution rate to the model and Spearman coefficient was eliminated.

Data of future climate were obtained from the Worldclim 2.0 database (Downloaded in July 2025) using the sixth coupled model (BCC-CSM2-MR) of the Coupled Model Intercomparison Project (CMIP6). The future climate was represented using three future periods, namely 2050s (year 2041–2060 average), 2070s (year 2061–2080 average), and 2090s (year 2081–2100 average). Considering the uncertainty of global climate change, three different Shared Socioeconomic Pathways are selected to represent three different future climate scenarios, namely SSP 126 (representing the minimum emission scenario), SSP 245 (representing the medium emission scenario), and SSP 585 (representing the maximum emission scenario) (Moss et al., 2010). For future climate projections (2050s, 2070s, 2090s), lumpy skin disease suitability was modeled using only bioclimatic variables under each SSP scenario; cattle and buffalo density was not included in future projections because reliable global projections of future livestock density under different climate and socioeconomic scenarios are not currently available at a comparable resolution. As a result, future projections represent climatic suitability for lumpy skin disease rather than full occurrence potential.

### 2.3 Establishment of the MaxEnt model of lumpy skin disease and *S. calcitrans*

The MaxEnt model of lumpy skin disease and *S. calcitrans* was established using MaxEnt software version 3.4.4. Background points (pseudo-absences) were randomly selected across the study area (global extent), with 10,000 background points used for each model run. All environmental layers were used at a spatial resolution of 2.5 arcminutes. Feature class options were set to “auto features” (the default), which allows MaxEnt to automatically select the appropriate feature types based on sample size. The regularization multiplier was set to the default value of 1.0. No model tuning was performed, and no bias file was used. The lumpy skin disease epidemic points and *S. calcitrans* distribution containing exact coordinates were selected as the sample set, and the filtered variables were selected as the environment variable set. Therein, 25% of the data was randomly selected as the test set, and the remaining 75% was utilized as the training set, and this was repeated 10 times (Li et al., 2025). The output forecast map could intuitively show the simultaneous suitable area of lumpy skin disease and *S. calcitrans*.

We divided “presence” or “absence” of lumpy skin disease and *S. calcitrans* distribution using maximum test sensitivity plus specificity (MTSPS) under future climate scenarios (Aidoo et al., 2022). To quantify changes in suitable areas under future climate scenarios, continuous suitability outputs were first converted to binary maps (suitable/unsuitable) using the maximum test sensitivity plus specificity (MTSPS) threshold. For each SSP scenario and time period, the binary future suitability map was compared with the binary current suitability map on a pixel-by-pixel basis. The area of each category was calculated in ArcGIS 10.8 by multiplying the number of pixels by the pixel area. The “unchanged” category in Table 1 includes both unsuitable retained and suitable retained pixels, while “increased” and “decreased” represent areas of range expansion and contraction, respectively. The lumpy skin disease suitable areas map was output by overlaying the lumpy skin disease climate suitability map with the vector distribution map, which represents the simultaneous suitable area of lumpy skin disease and its vectors (Peng et al., 2025). All the predicted lumpy skin disease suitable areas map were imported into ArcGIS 10.8 for visualization.

**Table 1:** Description of the environmental variables utilized in the MaxEnt model.

Variable	Description	Unit
Bio1	Annual mean temperature	°C
Bio2	Mean diurnal range	°C
Bio3	Isothermality	%
Bio4	Temperature seasonality	°C
Bio5	Max temperature of warmest month	°C
Bio6	Min temperature of coldest month	°C
Bio7	Temperature annual range	°C
Bio8	Mean temperature of wettest quarter	°C
Bio9	Mean temperature of driest quarter	°C
Bio10	Mean temperature of warmest quarter	°C
Bio11	Mean temperature of coldest quarter	°C
Bio12	Annual precipitation	mm
Bio13	Precipitation of wettest month	mm
Bio14	Precipitation of driest month	mm
Bio15	Precipitation seasonality	/
Bio16	Precipitation of wettest quarter	mm
Bio17	Precipitation of driest quarter	mm
Bio18	Precipitation of warmest quarter	mm
Bio19	Precipitation of coldest quarter	mm
CDD	Cattle distribution density	Head/km^2^
BDD	Buffalo distribution density	Head/km^2^

### 2.4 Evaluation and interpretation of the MaxEnt model

The jackknife test was utilized to determine the relative influence of each environmental variable on the results in the MaxEnt model, identifying the most important variable (Gao et al., 2020). The response curve was utilized to analyze the specific effect of each variable on the distribution results and the tendency. Multiple complementary indicators were adopted to comprehensively assess model performance, including the area under the Receiver Operating curve (AUC) to the x-axis, TSS, and test omission error rate were calculated to test and evaluate the performance of the MaxEnt model (Li et al., 2025). The value of AUC is generally between 0 and 1, and the higher values indicate greater accuracy (Ding et al., 2024). The performance of the model is good when the AUC value is greater than 0.8. The TSS was calculated as “Sensitivity+Specificity-1” based on the optimal threshold that maximizes test sensitivity plus specificity, which directly reflects the model’s ability to distinguish suitable habitats from unsuitable ones, with TSS > 0 representing predictive capacity superior to random guessing. The test omission refers to the proportion of real species occurrence points mistakenly classified as unsuitable habitat under the optimal threshold, and a low omission rate (<0.2) means few actual distribution sites are omitted. The average value of AUC, TSS and test omission were obtained after 10 repeated operations.

## 3 Results

### 3.1 Global lumpy skin disease distribution under future climate scenarios

After processing, a total of 2,591 lumpy skin disease occurrence records were retained. Seven environmental variables, Bio5, Bio12, Bio2, Bio4, Bio15, Bio17, and Bio19, were screened following multiple covariance tests. Besides, cattle distribution density and buffalo distribution density were also incorporated into the MaxEnt model for lumpy skin disease occurrence.

**Figure 2:**
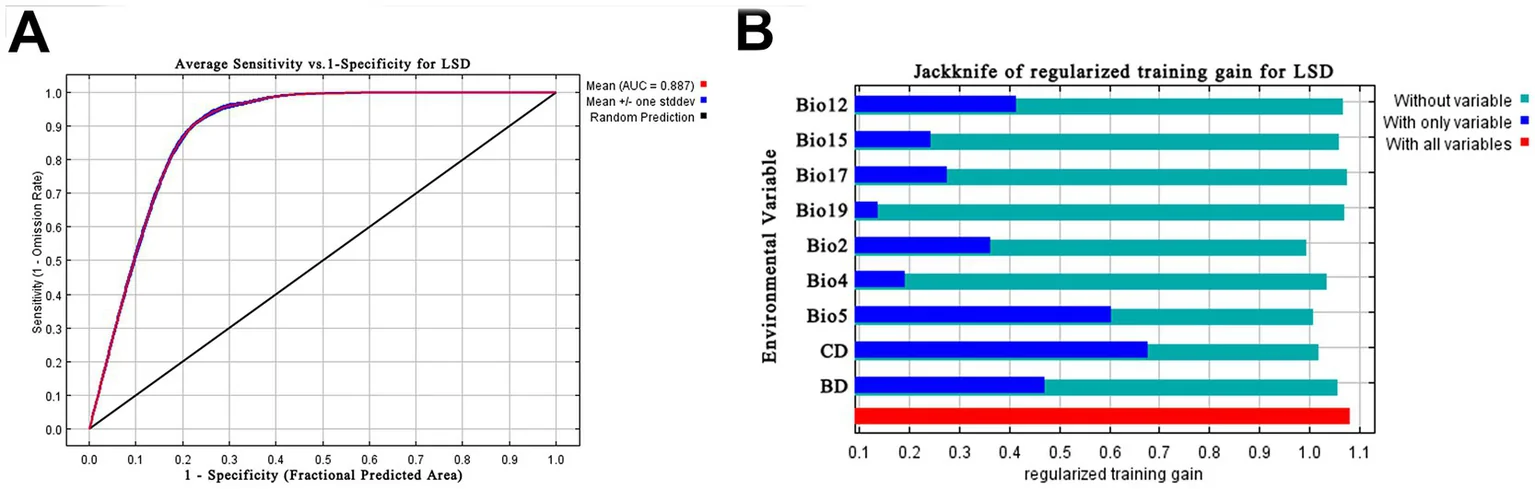
The area under the receiver operating curve (AUC) in the MaxEnt model for lumpy skin disease and the Jackknife test results of environmental variables. **(A)** The AUC in the MaxEnt model for lumpy skin disease; **(B)** the Jackknife test results of environmental variables.

As shown in Figure 2A, the average AUC value in the MaxEnt model for lumpy skin disease was 0.887 ± 0.004, indicating relatively high accuracy in predicting the environmental suitability for lumpy skin disease. The average TSS was 0.754 and the average test omission was 0.076. The TSS indicated that the model could effectively distinguish suitable habitats from unsuitable areas and the low omission suggested that the model hardly missed actual disease occurrence points. The jackknife test assessed the contribution of each variable, with results shown in Figure 2B. Among the nine variables, cattle distribution density (CDD), the max temperature of warmest month (Bio5), buffalo distribution density (BDD), and annual precipitation (Bio12) exhibited significantly higher contribution rates compared to the others, suggesting that these four variables were the important ones influencing the environmental suitability of lumpy skin disease.

**Figure 3:**
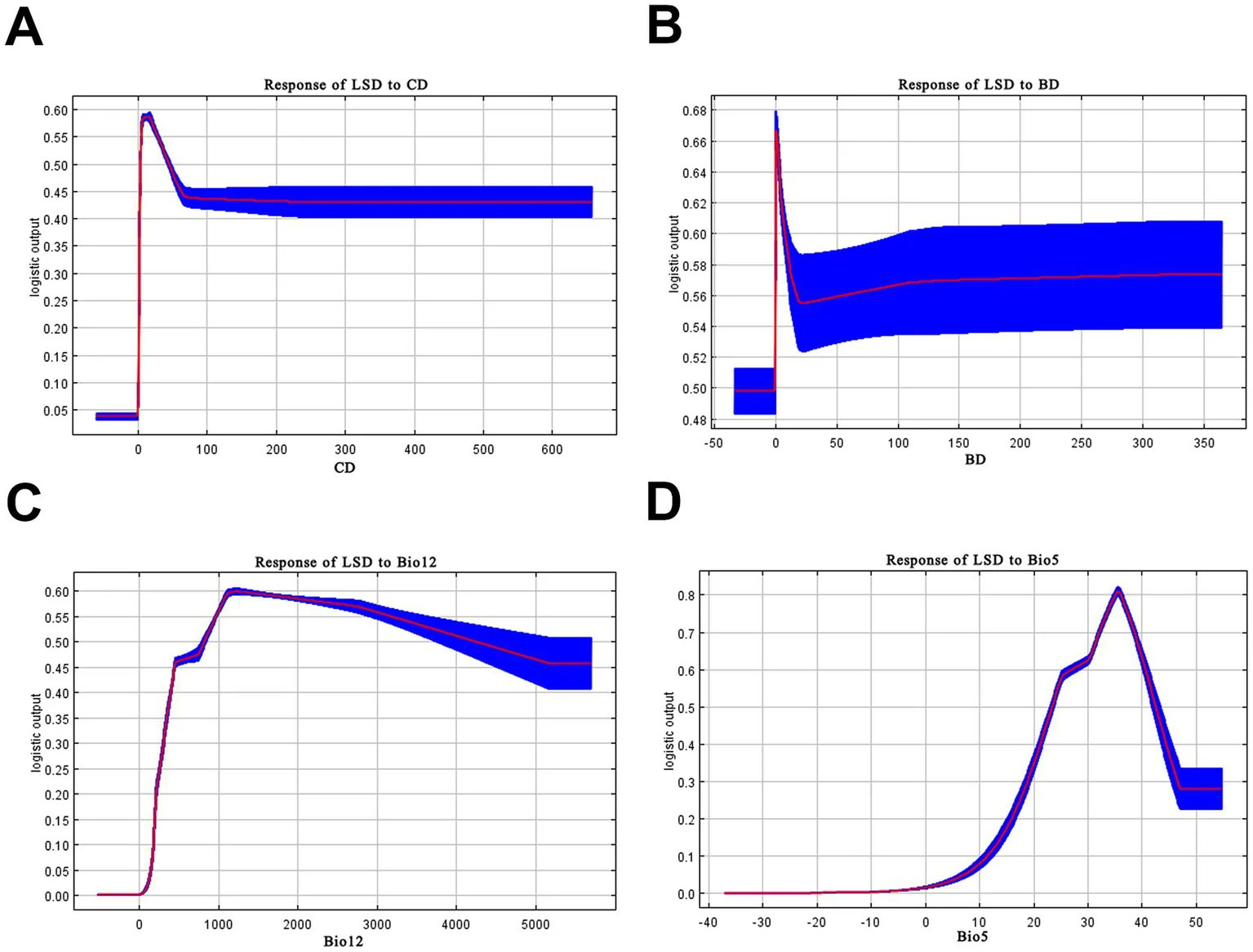
Response curves of four important environmental variables in the MaxEnt model for lumpy skin disease: **(A)** cattle distribution density (CDD); **(B)** buffalo distribution density (BDD); **(C)** annual precipitation (Bio12); **(D)** the maximum temperature of warmest month (Bio5).

The response curves of four important environmental variables on lumpy skin disease occurrence were presented in Figure 3. In general, the occurrence for lumpy skin disease increased with higher cattle distribution density (CDD) and buffalo distribution density (BDD). Annual precipitation (Bio12) between 500 mm and 5,000 mm relatively increased the occurrence of lumpy skin disease, with an optimal annual precipitation of approximately 1,000 mm. The maximum temperature of the warmest month (Bio5) between 10 °C and 45 °C was relatively conducive to the occurrence of the lumpy skin disease, with an optimal maximum temperature of about 35 °C.

**Figure 4:**
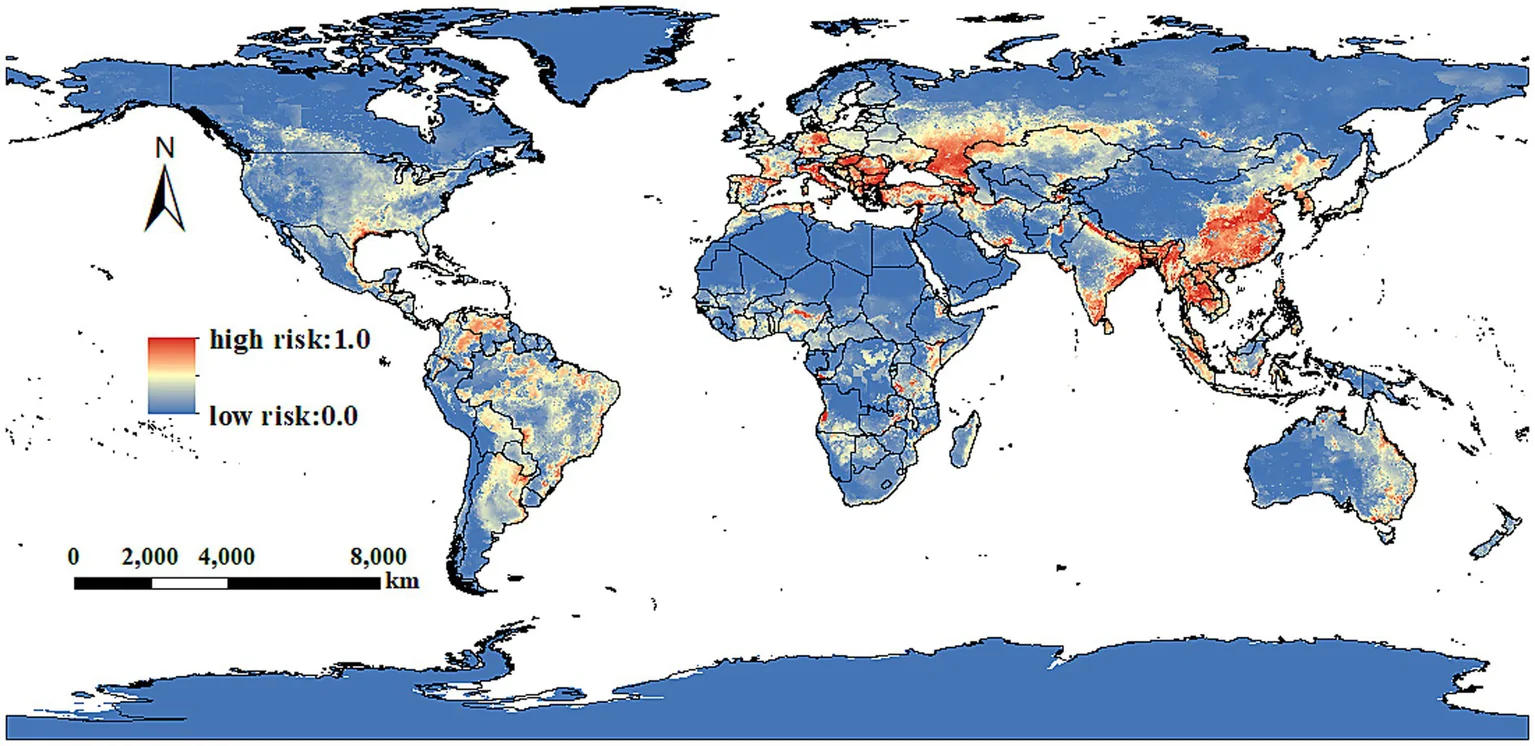
Prediction map of the current suitable areas of lumpy skin disease.

The prediction map (Figure 4) found that the high-suitable regions of lumpy skin disease include southern North America (southern Canada, central and eastern United States, and eastern Mexico), central and eastern South America (Venezuela, Colombia, Brazil, northern Argentina, and Bolivia), central and southern Europe, eastern and western Africa (including Nigeria, Tanzania, and Kenya), western Asia (southwestern Russia), southern Asia (India), southeast Asia (central and eastern China, Myanmar, Thailand, Laos, Vietnam, and Cambodia), and central and eastern Australia. The low-suitable regions of lumpy skin disease include northern North America (Alaska, northern Canada, Greenland), southern South America (southern Chile, southern Argentina), northern Europe (Norway, northern Sweden, Finland), the northern Saharan region of Africa (central-southern Algeria, central-southern Libya, central-northern Niger, northern Sudan), Saudi Arabia, northern and eastern Russia, western China, southwestern Australia, and Antarctica.

**Figure 5:**
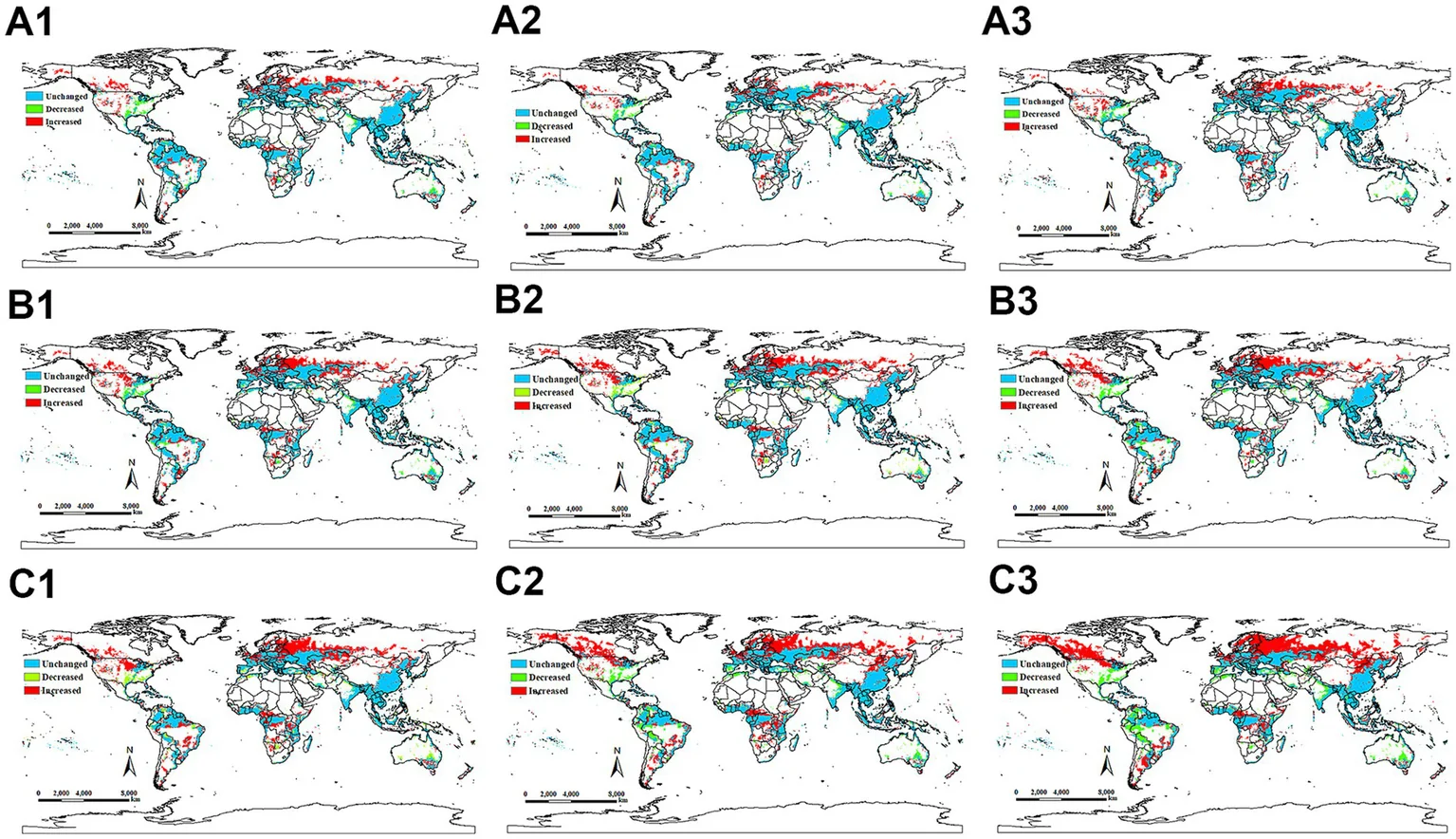
Prediction maps of suitable areas of lumpy skin disease under future climate scenarios **(A1)** SSP126- year 2050s; **(A2)** SSP126- year 2070s; **(A3)** SSP126- year 2090s; **(B1)** SSP245- year 2050s; **(B2)** SSP245- year 2070s; **(B3)** SSP245- year 2090s; **(C1)** SSP585- year 2050s; **(C2)** SSP585- year 2070s; **(C3)** SSP585- year 2090s. SSP126 represents the minimum emission scenario, SSP245 represents the medium emission scenario, and SSP585 represents the maximum emission scenario; year 2050s represent years from 2041 to 2060, year 2070s represent years from 2061 to 2080, year 2090s represent years from 2081 to 2100.

Under the three climate change scenarios, the suitable area for lumpy skin disease expanded to varying extents. The primary areas of expansion were concentrated in the Northern Hemisphere, specifically in mid- to high-latitude regions between approximately 40° and 70°N. Expansion in the Southern Hemisphere was limited to a small area. Conversely, contraction of suitable areas was minimal and primarily occurred in mid- to low-latitude regions, roughly between 20° and 40°N, encompassing the central and eastern United States, northern and central South America, North Africa, South Asia, and southern Australia (Figure 5).

As greenhouse gas emissions increased, the expansion of suitable areas for lumpy skin disease grew. Under the SSP245 scenario, the area of stable regions showed a slight increase compared to the SSP126 scenario in the year 2070s. Under the SSP126, SSP245, SSP585 scenarios, the area of expansion progressively increased over time, except for the SSP245 scenario in the year 2070s, where the expansion was slightly less than the year 2050s (Table 2).

**Table 2:** Changed in the area of suitable regions of lumpy skin disease under future climate scenarios (10^4^ km^2^).

Climate scenario (SSPs)	Changes in area (×10^4^ km^2^) of year 2050s (2041–2060 average)			Changes in area (×10^4^ km^2^) of year 2070s (2061–2080 average)			Changes in area (×10^4^ km^2^) of year 2090s (2081–2100 average)		
	Unchanged	Decreased	Increased	Unchanged	Decreased	Increased	Unchanged	Decreased	Increased
SSP126	3013.8	301.0	940.6	2910.5	282.8	838.8	2962.0	236.8	1184.2
SSP245	2938.9	293.4	1,255.4	3016.2	325.2	1489.8	2876.4	399.2	1424.9
SSP585	2728.9	351.0	1,641.3	2812.7	482.6	2075.7	2647.0	684.7	2519.5

SSP126 represents the minimum emission scenario, SSP245 represents the medium emission scenario, and SSP585 represents the maximum emission scenario. Areas were calculated by comparing binary suitability maps (MTSPS) between current and future periods. “Increased” indicates pixels that become suitable in the future (range expansion); “Decreased” indicates pixels that become unsuitable (range contraction); “Unchanged” includes pixels that remain either suitable or unsuitable in both periods.

### 3.2 Prediction of global *S. calcitrans* distribution under current climate scenario

**Figure 6:**
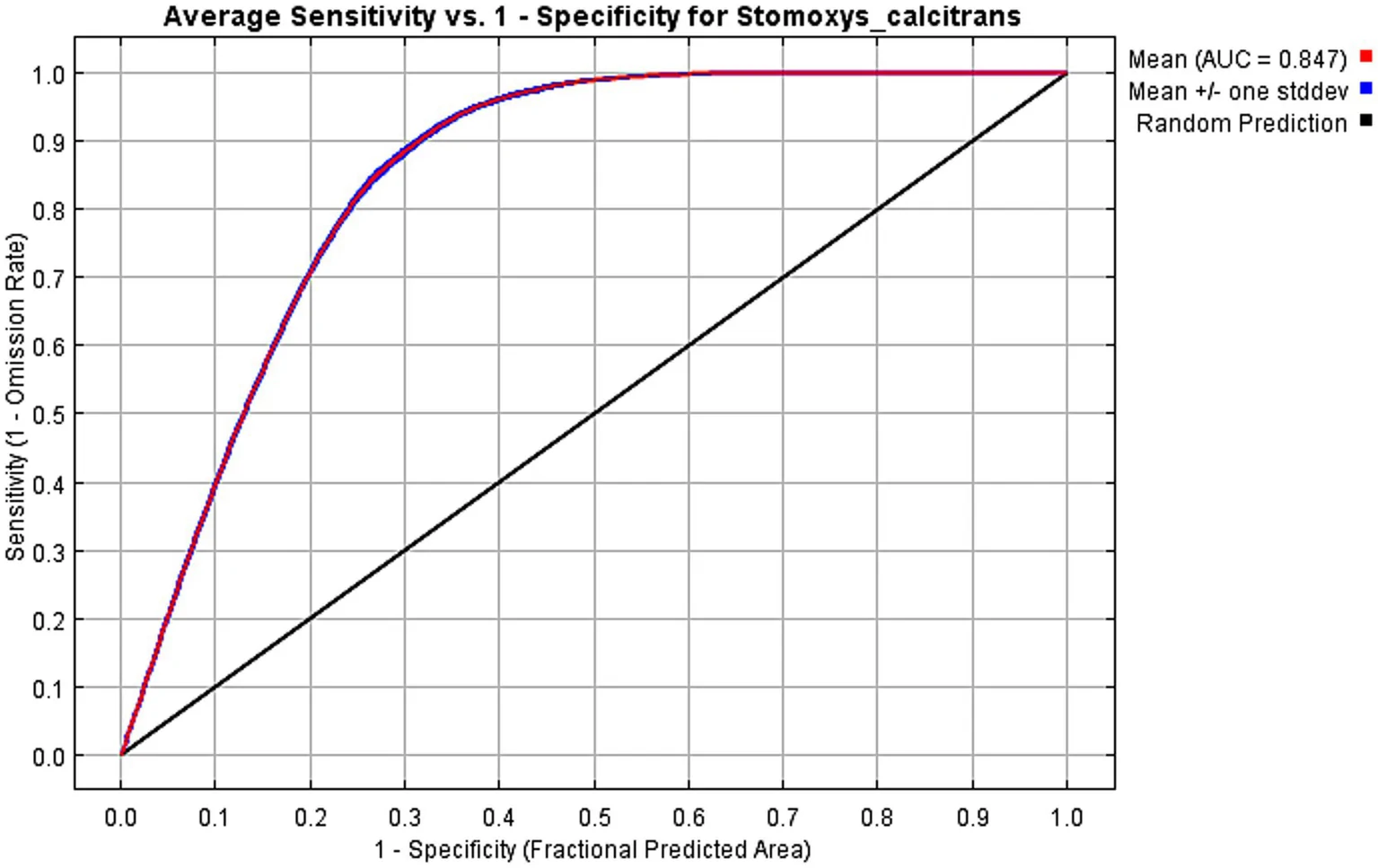
The AUC of MaxEnt for *Stomoxys calcitrans* distribution.

After data processing, 4,000 records of *S. calcitrans* distribution were retained. Seven bioclimatic variables, Bio14, Bio11, Bio12, Bio3, Bio7, Bio15, and Bio19, were screened following multiple covariance tests. As shown in Figure 6, the AUC value of the MaxEnt model for *S. calcitrans* distribution was 0.847 ± 0.004 after 10 repetitions. The average TSS was 0.686 and the average test omission was 0.098.

**Figure 7:**
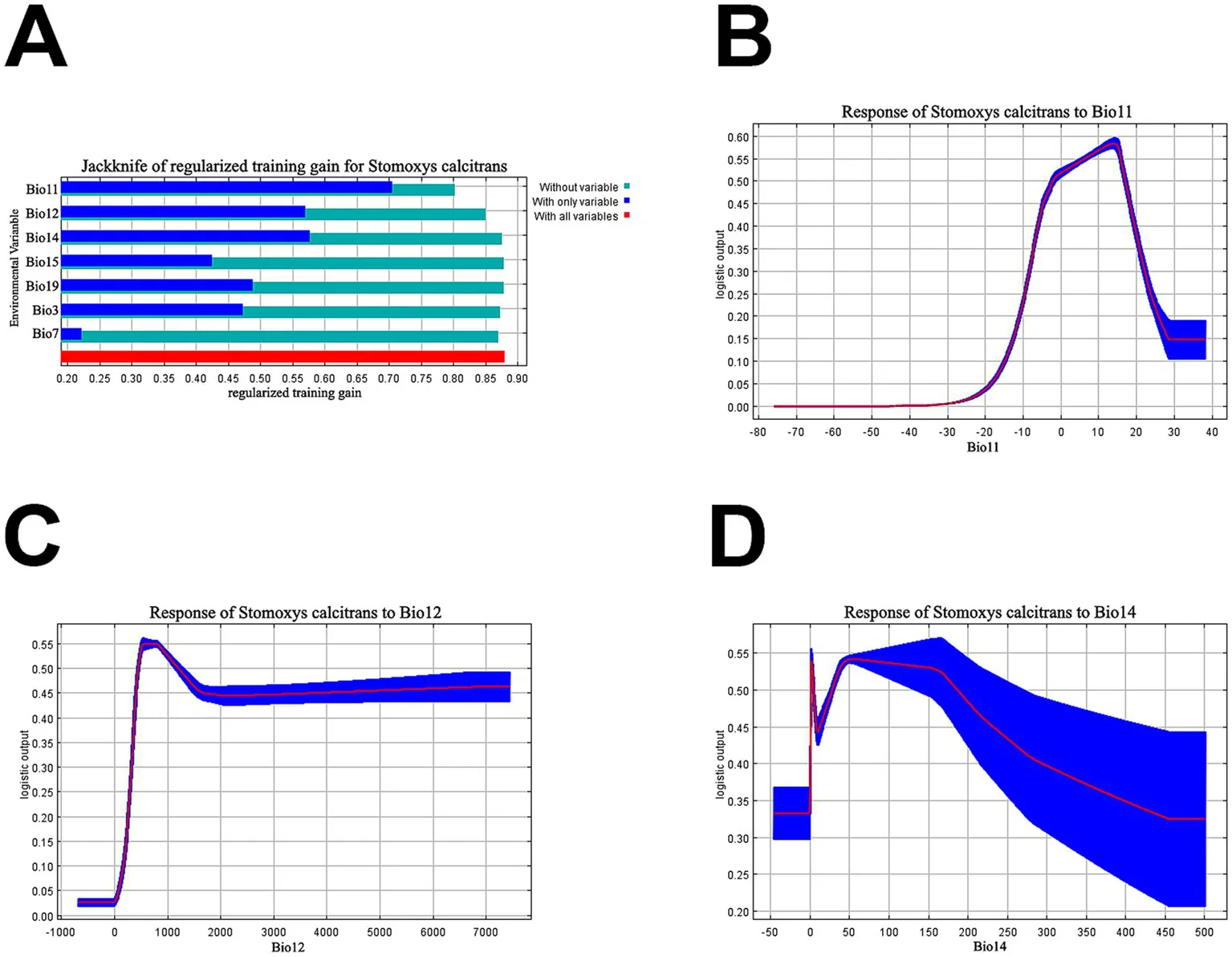
Jackknife test results of environmental variables and response curves of three key variables in MaxEnt for *Stomoxys calcitrans*. **(A)** Jackknife test results of environmental variables. **(B)** Response curve for the mean temperature of the coldest quarter (Bio11) of MaxEnt for *S. calcitrans*. **(C)** Response curve for annual precipitation (Bio12) of MaxEnt for *S. calcitrans*. **(D)** Response curve for precipitation of the driest month (Bio14) of MaxEnt for *S. calcitrans*.

The response curves depicted the effects of three key variables on *S. calcitrans* occurrence (Figure 7). The mean temperature of the coldest quarter (Bio11) was detrimental to *S. calcitrans* when below −15 °C or above 30 °C, with the optimal temperature around 15 °C. Insufficient annual precipitation (Bio12) hindered *S. calcitrans*, with the optimal annual precipitation being approximately 500 mm. Beyond 2,000 mm of annual precipitation, the suitability for *S. calcitrans* stabilized, showing minimal further change. The precipitation of the driest month (Bio14) also influenced *S. calcitrans* suitability, with too low precipitation being unfavorable. The optimal precipitation level for the driest month was around 150 mm.

**Figure 8:**
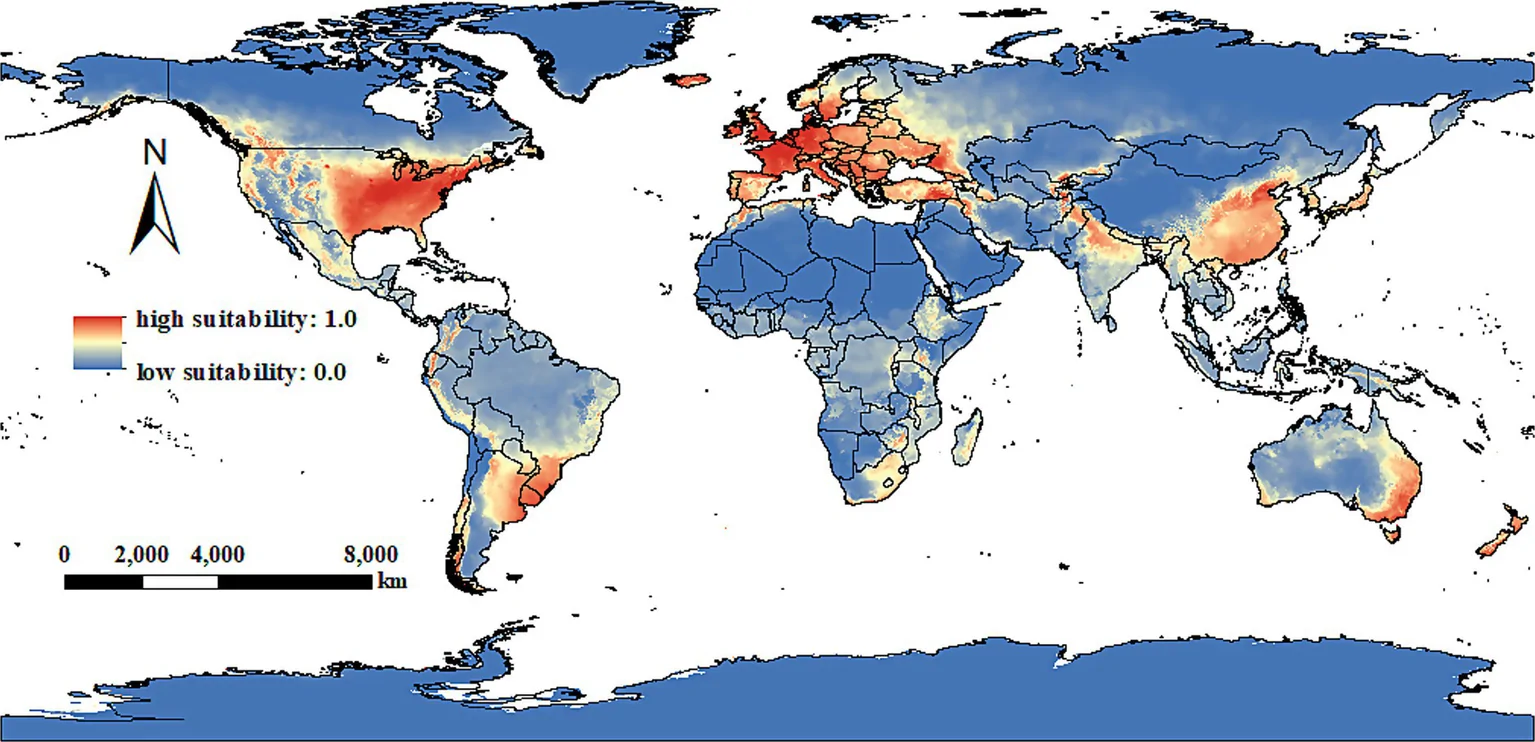
Prediction map of current environmental suitability of *Stomoxys calcitrans*.

As shown in Figure 8, the distribution map of *S. calcitrans* reveals that all regions, except Antarctica, northeastern North America, and most of northeastern Asia, had potential habitat conditions for the vector. High vector suitability was found in eastern North America (eastern United States), southeastern South America (southern Brazil, eastern Argentina, and Uruguay), Europe, East Asia (eastern China), Southwest Asia (northern India and northeastern Pakistan), and eastern Australia. Low suitability was observed in Northern North America (Alaska, northern Canada, Greenland), central South America (central Argentina), the northern Amazon rainforest region of South America (northern Brazil, southern Colombia, southern Venezuela), central-western Africa (southern Algeria, central-eastern Mali, southern Niger, southern Chad, southern Egypt, northeastern Sudan), central-eastern Russia, and Antarctica.

### 3.3 Global lumpy skin disease occurrence suitable areas

**Figure 9:**
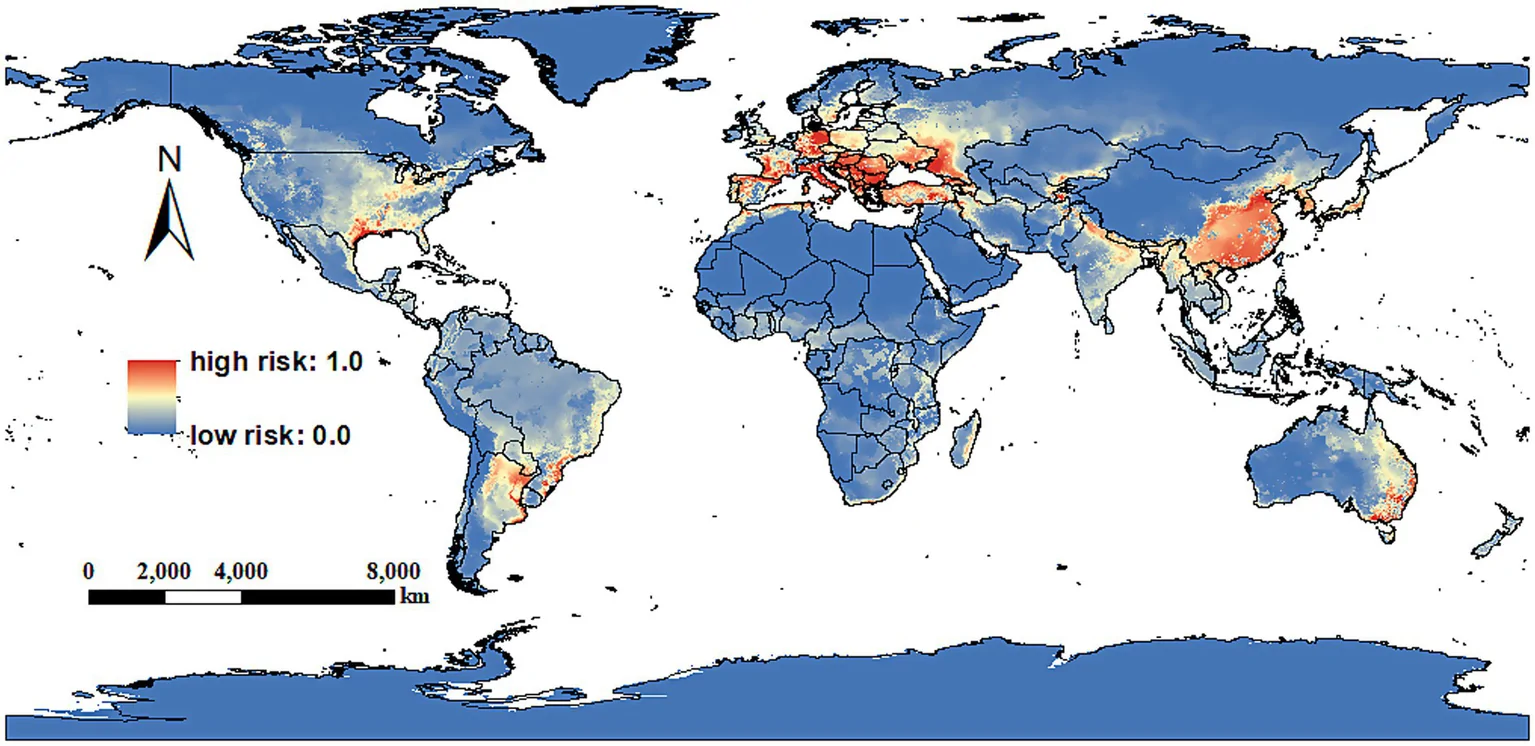
Suitable areas map of lumpy skin disease occurrence.

The suitable areas map for lumpy skin disease occurrence, shown in Figure 9, indicates regions where both high climatic suitability for lumpy skin disease and high vector distribution suitability overlap. High-suitable areas include the south-central United States, northeastern Argentina, southeastern Brazil, Europe (excluding northern Europe, such as Iceland, Norway, and Sweden), the Mediterranean coastal areas of North Africa (such as Tunisia, northern Algeria, and Morocco), Turkey, Tajikistan, northern India, eastern China, South Korea, central and southern Japan, northern Indochina, and eastern Australia. Low-suitable areas for lumpy skin disease occurrence include Alaska, United States, the central and northern Canada, Greenland, northern Chile, the Sahara region in Africa, northern regions such as Siberia in Russia.

## 4 Discussion

In this study, the MaxEnt model was developed utilizing lumpy skin disease outbreak data, *S. calcitrans* distribution points, and environmental variables to assess the global suitable areas of lumpy skin disease outbreaks. This study provides valuable guidance and reference for future research based on the latest data on lumpy skin disease and *S. calcitrans*. The findings will assist countries in implementing targeted surveillance and prevention strategies, thereby mitigating the suitable areas of outbreaks and reducing associated economic losses.

Cattle and buffalo are the primary hosts susceptible to lumpy skin disease (Gilbert et al., 2018). Their distribution density directly influences the likelihood of outbreaks, with cattle contributing 58.2% and buffalo 14.9% to the model. The response curves revealed that the probability of an outbreak generally increased with the density of these animals, although a negative correlation was observed at low densities, likely due to well-managed breeding practices or geographical barriers such as mountain ranges. High densities of susceptible animals promote vector breeding, as *S. calcitrans* preferentially targets large-scale cattle herds (Semelbauer et al., 2018), where lumpy skin disease spreads rapidly. Intensive farming practices that increase herd mobility and introduce exotic breeds further elevate the risk of outbreaks (Tuppurainen and Oura, 2012).

The MaxEnt model also highlighted the impact of current climatic conditions on lumpy skin disease occurrence. Previous studies have indicated that mean temperature and precipitation are key variables influencing LSDV distribution (Alkhamis and VanderWaal, 2016; Machado et al., 2019), which aligns with the findings of this study. The response curve for the maximum temperature of the warmest month (Bio5) showed that temperatures below 10 °C or above 45 °C were unfavorable for lumpy skin disease occurrence. Low temperatures reduce vector activity and the efficiency of virus transmission (Gilles et al., 2005b), while suitable overwintering conditions, such as moderate temperatures, favor the survival of LSDV through transmission *via* vector eggs and larvae (Kumar et al., 2025). Conversely, excessively high temperatures increase water evaporation and exacerbate drought conditions, which negatively impact the survival and reproductive capabilities of vectors (Ardestani and Mokhtari, 2020). Precipitation plays a pivotal role in influencing the activity levels of bloodsucking insects (Robert et al., 2020). Studies have indicated that the widespread prevalence of lumpy skin disease is linked to high rainfall and increased insect activity (Ochwo et al., 2019). The response curve for annual precipitation (Bio12) revealed that precipitation levels below 500 mm or above 5,000 mm hinder the occurrence of lumpy skin disease, with the optimal value around 1,000 mm. This suggests that lumpy skin disease epidemics are more likely to occur under conditions of moderate precipitation. Ochwo et al. (2019) observed that regions with more than 1,000 mm of annual precipitation are more prone to lumpy skin disease outbreaks. In the present study, the emergence of lumpy skin disease peaked at approximately 1,000 mm of annual precipitation (Bio12), correlating with higher probabilities of *S. calcitrans* emergence. This highlights that precipitation influences the likelihood of lumpy skin disease by affecting vector activity levels. Molla et al. (2017) findings indicated that outbreaks of lumpy skin disease typically peak in October, suggesting that higher rainfall promotes the reproduction and survival of bloodsucking insects, facilitating the spread of LSDV. Furthermore, sufficient precipitation can lead to the formation of shared irrigation and grazing water sources, which increases direct contact between cattle and elevates the suitable areas of transmission.

This study revealed distinct geographical patterns for lumpy skin disease under current climate conditions. Under future climate scenarios, the suitable area for the disease continues to expand, predominantly moving to mid- to high-latitude regions. This trend is likely a consequence of global warming driven by rising carbon emissions (Luo et al., 2024), which increases temperatures in northern regions, creating conditions conducive to vector survival and LSDV persistence. Consequently, lumpy skin disease is expected to spread to mid- to high-latitude regions. This study suggests that the suitable areas of lumpy skin disease will persist in the global South while gradually rising in the North. Livestock farming in high-latitude countries may face greater threats. Such shifts in the ecological niche of lumpy skin disease due to climate change could significantly increase the suitable areas of arboviral infections worldwide.

Temperature and precipitation are key factors driving seasonal variations in *S. calcitrans* populations (Taylor et al., 2017) and influencing their population density (Taylor et al., 2007; Semelbauer et al., 2018). The population size of *S. calcitrans* peaks in July in northeastern Kansas, United States (Doud et al., 2012), and coincides with the rainy season (Phetcharat et al., 2024). Changes in *S. calcitrans* populations are positively correlated with precipitation levels in the preceding 1–2 weeks and temperature during the same week (Taylor et al., 2007). This study revealed that precipitation in the driest month (Bio14) contributed 31.9% to the model, with the distribution probability of *S. calcitrans* peaking when precipitation in the driest month was around 150 mm. Similarly, higher annual precipitation (Bio12) tends to favor the occurrence of lumpy skin disease. A reduction in precipitation, however, results in a decline in *S. calcitrans* populations during summer months (Taylor et al., 2007). *S. calcitrans* lays more eggs in moist, sandy soils (Baleba et al., 2019), indicating that larvae prefer humid conditions. Thus, moderate precipitation provides an ideal humid environment for the larvae and eggs, while excessive rainfall hinders larval development. Temperature also plays a significant role in shaping the potential distribution of *S. calcitrans* (Broce et al., 2005; Doud et al., 2012). Preferring humid, sunny environments, *S. calcitrans* larvae cannot survive at temperatures above 35 °C (Florez-Cuadros et al., 2019), and adults have a longer lifespan at 20 °C (Gilles et al., 2005a). High summer temperatures reduce *S. calcitrans* fecundity, leading to a population decline (Semelbauer et al., 2018). In the present study, the mean temperature of the coldest season (Bio11) contributed 28% to the model, with the distribution probability of *S. calcitrans* peaking at temperatures between 10 °C and 20 °C. Survival rates for *S. calcitrans* were unfavorable when the mean temperature of the coldest season fell below −10 °C or exceeded 30 °C. Gilles found that *S. calcitrans* larvae survive well at temperatures between 20 and 25 °C, with low temperatures causing larval mortality and delayed development, while high temperatures lead to pupal mortality (Gilles et al., 2005b). These findings align with the results of this study that *S. calcitrans* distribution is concentrated in warmer regions, such as the southeastern United States and the southern Mediterranean coast of Europe. These areas, which also coincide with the ones most likely to transmit lumpy skin disease, exhibit a higher suitable areas of disease outbreaks.

The lumpy skin disease suitable areas map was output by overlaying the lumpy skin disease climate suitability map with the vector distribution map, which represents the maximum probability of the simultaneous presence of both suitable climatic conditions for lumpy skin disease and the existence of vectors. High-suitable areas not only meet the environmental suitability requirements for lumpy skin disease but also have relatively high vector densities, creating favorable conditions for disease transmission. Once LSDV enters these high-suitable zones, a complete transmission chain is likely to form, leading to potential outbreaks and epidemics. This would result in significant morbidity in large ruminants, such as buffalo and cattle, causing substantial economic losses. The prediction results identified southern Europe, Southeast Asia, and eastern Australia as high-suitable regions, suggesting that the climatic conditions (precipitation and temperature) in these areas are conducive to the survival and transmission of *S. calcitrans* and LSDV, making them prone to lumpy skin disease epidemics.

This study also has limitations. First, our study assesses environmental suitability and occurrence potential rather than comprehensive epidemiological risk. True disease risk is determined by a complex interplay of multiple factors beyond climate and host density, including animal movement and trade patterns, vaccination coverage, herd management practices, biosecurity measures, veterinary service capacity, disease reporting infrastructure, control policies, and historical outbreak dynamics. Our models focus on the environmental dimension of disease potential, which is a necessary but not sufficient component of comprehensive risk assessment. Second, the MaxEnt model primarily utilized bioclimatic variables and host density variables, without considering the influence of additional factors such as human activities, land use changes, and livestock management practices. These factors may also affect the distribution and transmission of lumpy skin disease and its vector. Thirdly, several sources of uncertainty should be considered when interpreting future projections. We used a single GCM (BCC-CSM2-MR) rather than a multi-model ensemble; while this model performs well for climate simulation, it does not capture full structural uncertainty across GCMs. Our projections also assume niche conservatism, climatic equilibrium, and unlimited dispersal. Despite these limitations, consistent directional trends across three SSP scenarios (low to high emissions) suggest our broad conclusions about range shifts are robust. Multi-GCM ensembles and dispersal modeling represent important future improvements.

An additional limitation of this study relates to potential sampling and reporting bias in the occurrence data. Lumpy skin disease outbreak records from EMPRES-i may be influenced by cross-national variation in surveillance capacity, disease awareness, reporting systems, and veterinary infrastructure, which could lead to underreporting in regions with less developed surveillance systems. Similarly, *S. calcitrans* records from GBIF are subject to spatial bias, with records tending to accumulate in well-studied, accessible, or densely sampled regions, while remote or less-studied areas may be underrepresented. While spatial thinning was applied to reduce spatial autocorrelation and clustering, this procedure does not fully correct for sampling bias, as it removes clustered records but does not address systematic under- or over-sampling across regions. We did not apply bias correction in our models, which means that the predicted suitable areas may partially reflect sampling effort rather than true environmental suitability in some regions. However, given the global extent of our study and the relatively broad geographic coverage of both datasets after thinning, we expect this bias to primarily affect predictions at local scales rather than the global patterns and key environmental drivers identified in our analysis. Future studies could incorporate bias files or target-group background approaches to further account for sampling heterogeneity.

Next, Future studies could focus on collecting more detailed data records to enable more precise predictions of distribution changes in lumpy skin disease and *S. calcitrans*, as well as integrating epidemiological and socioeconomic data to develop a more comprehensive risk assessment framework.

## 5 Conclusion

In this study, the MaxEnt model was constructed using global lumpy skin disease outbreak data and *S. calcitrans* distribution data. The climatic suitability maps for lumpy skin disease and *S. calcitrans* were combined to generate a global suitable areas map for lumpy skin disease, where environmental conditions may support both the disease and its vector. The results revealed that the environmental variables most influencing the suitable areas of lumpy skin disease included buffalo distribution density, cattle distribution density, the maximum temperature of the warmest month, and annual precipitation. Under the future climate scenarios, the expansion of climatic suitable areas for lumpy skin disease is expected to occur predominantly in mid- to high-latitude regions between 40° and 70°. High-suitable areas for lumpy skin disease were included the south-central United States, northeastern Argentina, southeastern Brazil, Europe (excluding northern Europe, such as Iceland, Norway, and Sweden), the Mediterranean coastal areas of North Africa (such as Tunisia, northern Algeria, and Morocco), Turkey, Tajikistan, northern India, eastern China, South Korea, central and southern Japan, northern Indochina, and eastern Australia. These projections represent environmental suitability only and do not predict actual disease occurrence. Lumpy skin disease emergence depends on additional epidemiological factors including animal movement, trade, vaccination, and surveillance. This study enhances the understanding of lumpy skin disease and provides scientific support for the development of more effective prevention and control strategies in regions with high environmental suitability.

## Data Availability

The original contributions presented in the study are included in the article/Supplementary material, further inquiries can be directed to the corresponding author/s.

## Footnotes

1 https://www.gbif.org/, Downloaded in July 2025.

2 http://www.worldclim.org, Downloaded in July 2025.

3 http://www.fao.org/livestock-systems/global-distributions/en/, Downloaded in July 2025.
