# Supplementary material for: MaxEnt-based suitable areas analysis of global lumpy skin disease under current and future climate scenarios
Source: Front Microbiol. 2026 Sep 7;17:1816805. doi: 10.3389/fmicb.2026.1816805 (PMC13590795; doi:10.3389/fmicb.2026.1816805)
Supplement: Supplementary file 4 [file Table_1.doc]

**Supplementary Table Important metrics of the environmental variables in the MaxEnt model**

| Variable | the MaxEnt model of lumpy skin disease | | | | | the MaxEnt model of *Stomoxys calcitrans* | | | | |
| --- | --- | --- | --- | --- | --- | --- | --- | --- | --- | --- |
|  | Max pairwise \|r\| | Most correlated variable | Contributions  (%) | Retained (Yes or No) | Reason | Max pairwise \|r\| | Most correlated variable | Contributions  (%) | Retained (Yes or No) | Reason |
| Bio1 | 0.9674 | Bio5 | 11.7 | N | Collinear with Bio5, lower contribution | 0.9803 | Bio11 | 4.9 | N | Collinear with Bio11, lower contribution |
| Bio2 | 0.6559 |  | 13.9 | Y |  | 0.6559 |  | 0 | N | 0 contribution |
| Bio3 | 0.8528 | Bio4 | 4.6 | N | Collinear with Bio4, lower contribution | 0.7908 |  | 4.7 | Y |  |
| Bio4 | 0.8528 | Bio3 | 5.1 | Y | Collinear with Bio3, Higher contribution | 0.9572 | Bio7 | 0.1 | N | Collinear with Bio7, lower contribution |
| Bio5 | 0.9973 | Bio10 | 22.9 | Y | Collinear with Bio10, Higher contribution | 0.9973 | Bio10 | 0.3 | N | Collinear with Bio10, lower contribution |
| Bio6 | 0.9979 | Bio11 | 2.5 | N | Collinear with Bio11, lower contribution | 0.9979 | Bio11 | 1.9 | N | Collinear with Bio11, lower contribution |
| Bio7 | 0.9572 | Bio4 | 1.5 | N | Collinear with Bio4, lower contribution | 0.7352 |  | 0.9 | Y |  |
| Bio8 | 0.9703 | Bio5 | 0.1 | N | Collinear with Bio5, lower contribution | 0.9762 | Bio10 | 0 | N | 0 contribution |
| Bio9 | 0.827 | Bio5 | 0.7 | N | Collinear with Bio5, lower contribution | 0.9555 | Bio11 | 0.4 | N | Collinear with Bio11, lower contribution |
| Bio10 | 0.9973 | Bio5 | 2.5 | N | Collinear with Bio5, lower contribution | 0.9156 | Bio11 | 1.3 | N | Collinear with Bio11, lower contribution |
| Bio11 | 0.9013 | Bio5 | 10.1 | N | Collinear with Bio5, lower contribution | 0.9979 | Bio6 | 28 | Y | Collinear with Bio6, Higher contribution |
| Bio12 | 0.937 | Bio16 | 17.9 | Y | Collinear with Bio16, Higher contribution | 0.9125 | Bio13 | 22.8 | Y | Collinear with Bio13, Higher contribution |
| Bio13 | 0.9929 | Bio16 | 0 | N | 0 contribution | 0.9125 | Bio12 | 0 | N | 0 contribution |
| Bio14 | 0.9927 | Bio17 | 0.3 | N | Collinear with Bio17, lower contribution | 0.9927 | Bio17 | 31.9 | Y | Collinear with Bio17, Higher contribution |
| Bio15 | 0.4847 |  | 2.6 | Y |  | 0.4847 |  | 0.9 | Y |  |
| Bio16 | 0.937 | Bio12 | 0.1 | N | Collinear with Bio12, lower contribution | 0.937 | Bio12 | 0.9 | N | Collinear with Bio12, lower contribution |
| Bio17 | 0.9927 | Bio14 | 1.3 | Y | Collinear with Bio14, Higher contribution | 0.9927 | Bio14 | 0.1 | N | Collinear with Bio14, lower contribution |
| Bio18 | 0.8353 | Bio12 | 1.1 | N | Collinear with Bio12, lower contribution | 0.8353 | Bio12 | 0.2 | N | Collinear with Bio12, lower contribution |
| Bio19 | 0.7612 |  | 1 | Y |  | 0.7612 |  | 0.6 | Y |  |

Note: Max pairwise |r| represents the maximum absolute Spearman correlation coefficient between each variable and any other initial predictor. Variable pairs with |r| ≥ 0.8 were considered collinear; for each collinear pair, the variable with higher percent contribution in the preliminary MaxEnt model was retained, and the variable with lower contribution was excluded.
